# Supplementary material for: Structural Properties of Phenylalanine-Based Dimers Revealed Using IR Action Spectroscopy
Source: Molecules. 2022 Apr 6;27(7):2367. doi: 10.3390/molecules27072367 (PMC9000879; doi:10.3390/molecules27072367)
Supplement: Supplementary file 1 [file molecules-27-02367-s001.zip › molecules-1616620-supplementary.pdf]

molecules

Supplementary Materials for Article

Structural Properties of Phenylalanine-Based Dimers Revealed Using IR Action Spectroscopy

Iuliia Stroganova [1,2], Sjors Bakels [1,2] and Anouk M. Rijs [1,*]

1 Division of BioAnalytical Chemistry, AIMMS Amsterdam Institute of Molecular and Life Sciences, Vrije Universiteit Amsterdam, De Boelelaan 1108, 1081 HV Amsterdam, The Netherlands.
2 Radboud University, FELIX Laboratory, Institute for Molecules and Materials, Toernooiveld 7, 6525 ED Nijmegen, The Netherlands.
* Correspondence: a.m.rijs@vu.nl

List of supplementary materials:

1. REMPI spectra of the PhgPhg monomer and dimer and IR-UV hole-burning spectrum of the PhgPhg monomer...... 2

2. Theoretical IR spectra of the structural families of the PhgPhg monomer 3

3. Theoretical IR frequencies characteristic for the PhgPhg monomer 4

4. Theoretical IR spectra of the antiparallel family of the PhgPhg dimer and their structures 5

5. Theoretical IR frequencies characteristic for the PhgPhg dimer 7

6. REMPI spectra of the FF monomer and dimer and IR-UV hole-burning spectrum of the FF monomer 8

7. The structural families of the FF monomer and their IR spectra 9

8. Theoretical IR frequencies characteristic for the FF dimer 12

9. Theoretical IR spectra of the structural families of the FF dimer 13

10. REMPI spectrum of the FFF dimer 15

11. Possible structure of the FFF dimer 16

12. XYZ files of assigned structures 17

13. References 21

1. REMPI spectra of the PhgPhg monomer and dimer and IR-UV hole-burning spectrum of the PhgPhg monomer

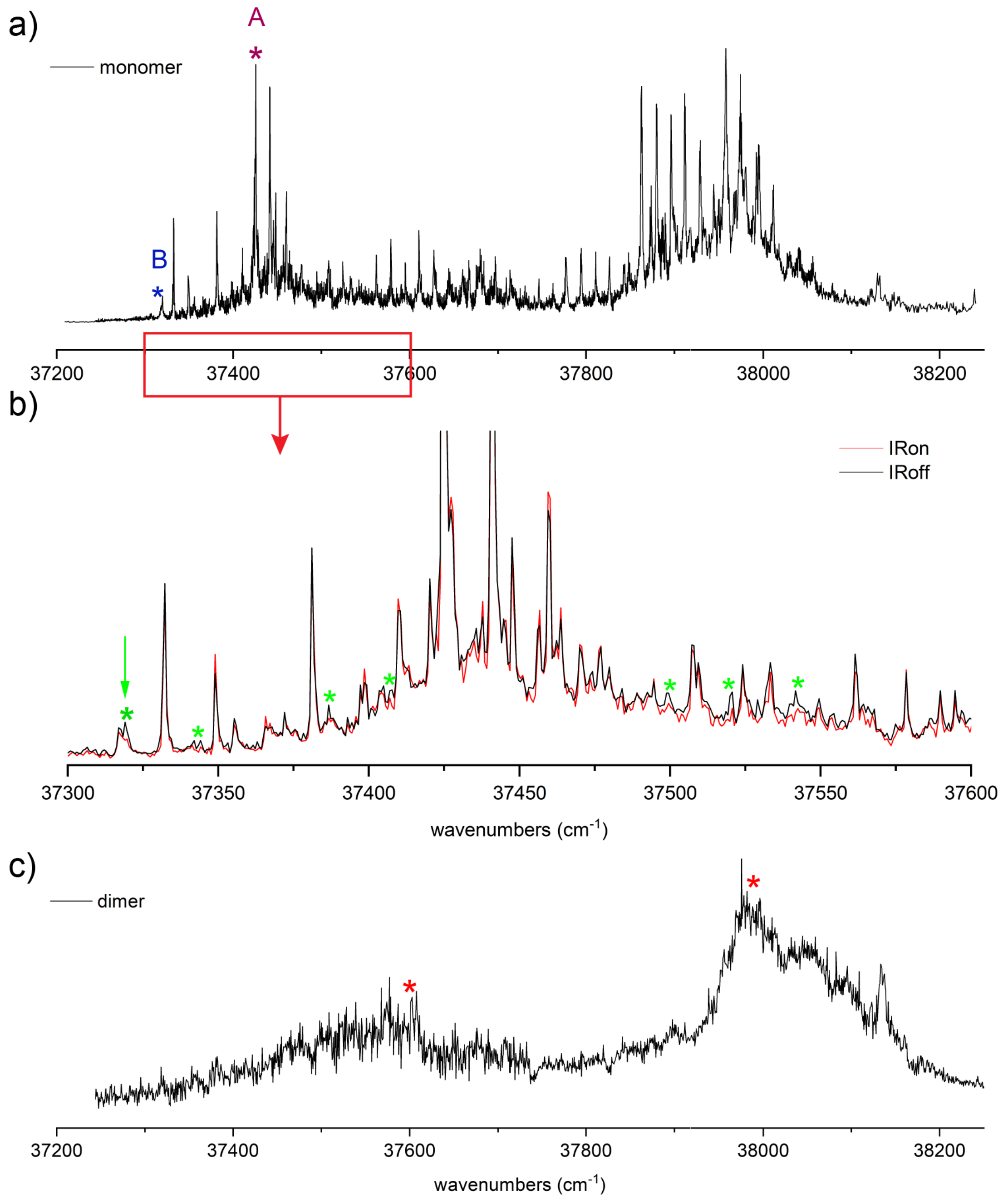

Figure S1: REMPI spectra of the PhgPhg monomer and dimer and IR-UV hole-burning spectrum of the PhgPhg monomer**:** **(a)** REMPI spectrum of the PhgPhg monomer. The IR spectrum of conformer A was recorded at 37426 cm^{-1} (purple asterisk) and of conformer B at 37319 cm^{-1} (blue asterisk). (**b**) IR-UV hole-burning spectrum of the PhgPhg monomer between 37300–37600 cm^{-1} as is indicated by the red box in **(a)**. The IR laser was set at 3426 cm^{-1} corresponding to the unique vibrational transition of conformer B. The green asterisks indicate the depletion in the UV signal meaning that these transitions correspond to conformer B. The green arrow shows the UV wavenumber that was selected to record the IR spectrum of conformer B. **(c)** REMPI spectrum of the PhgPhg dimer. The IR spectrum of the PhgPhg dimer was obtained at 37603 cm^{-1} and 37988 cm^{-1} (red asterisks).

2. Theoretical IR spectra of the structural families of the PhgPhg monomer

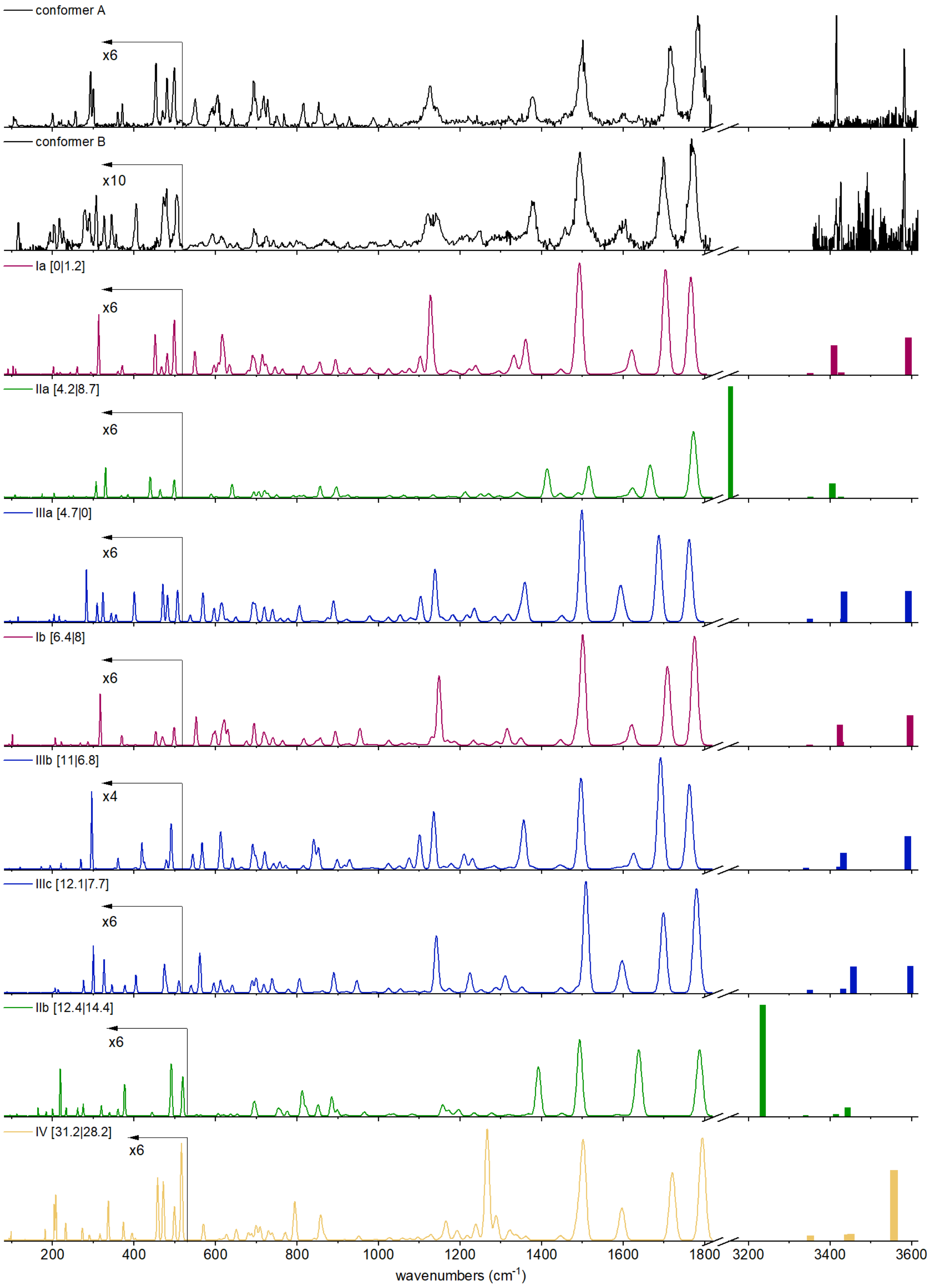

Figure S2: Theoretical IR spectra of the structural families of the PhgPhg monomer. Experimental (black, top) and calculated (colored) IR spectra of the families of PhgPhg monomers, arranged from lowest energy to highest (top to bottom). Calculated spectra are scaled by 0.976 (1800-100 cm^{-1}) and 0.956 (3100-3600 cm^{-1}), calculations have been done at the B3LYP-D3/6-311+G(d,p) level, their zero-point energies and Gibbs free energies at 300 K in kJ/mol are shown between square brackets. The region below 520 cm^{-1} was multiplied for clarity (multiplication factor indicated).

3. **Theoretical IR frequencies characteristic for the PhgPhg monomer**

Table S1: Experimentally observed (bold) and calculated frequencies in the amide I and II region for the PhgPhg monomer[1]

**Structure**	**Carboxylic C=O stretch**	**Peptide C=O stretch**	**NH bend**	**NH_2 scissoring**
Ia	1768 C5	1704 free	1493 2XC5	1621 -NH
IIa	1773 free	1669 C7	1515 C5 -NH_2	1622 -NH
IIIa	1763 C5	1686 C5 -NH_2	1499 C5 C=O	1596 -CO
Ib	1775 free	1708 free	1502 2XC5 -OH	1620 -NH
IIIb	1763 C5	1690 free	1498 C5 C=O	1625 free
IIIc	1780 free	1698 C5 -NH_2	1509 C5 -OH	1599 -CO
IIb	1788 free	1639 C5 -NH_2 C7	1493 π-interactions	1630 -CO weak
IV	1794 free	1720 C5 -NH_2	1502 C5 -OH	1597 -CO
**Conformer A**	**1783**	**1717**	**1497**	**1600**
**Conformer B**	**1770**	**1700**	**1495**	**1601**

[1]Frequencies are given in cm^{-1}. Calculated frequencies are scaled by 0.976.

4. Theoretical IR spectra of the antiparallel family of the PhgPhg dimer and their structures

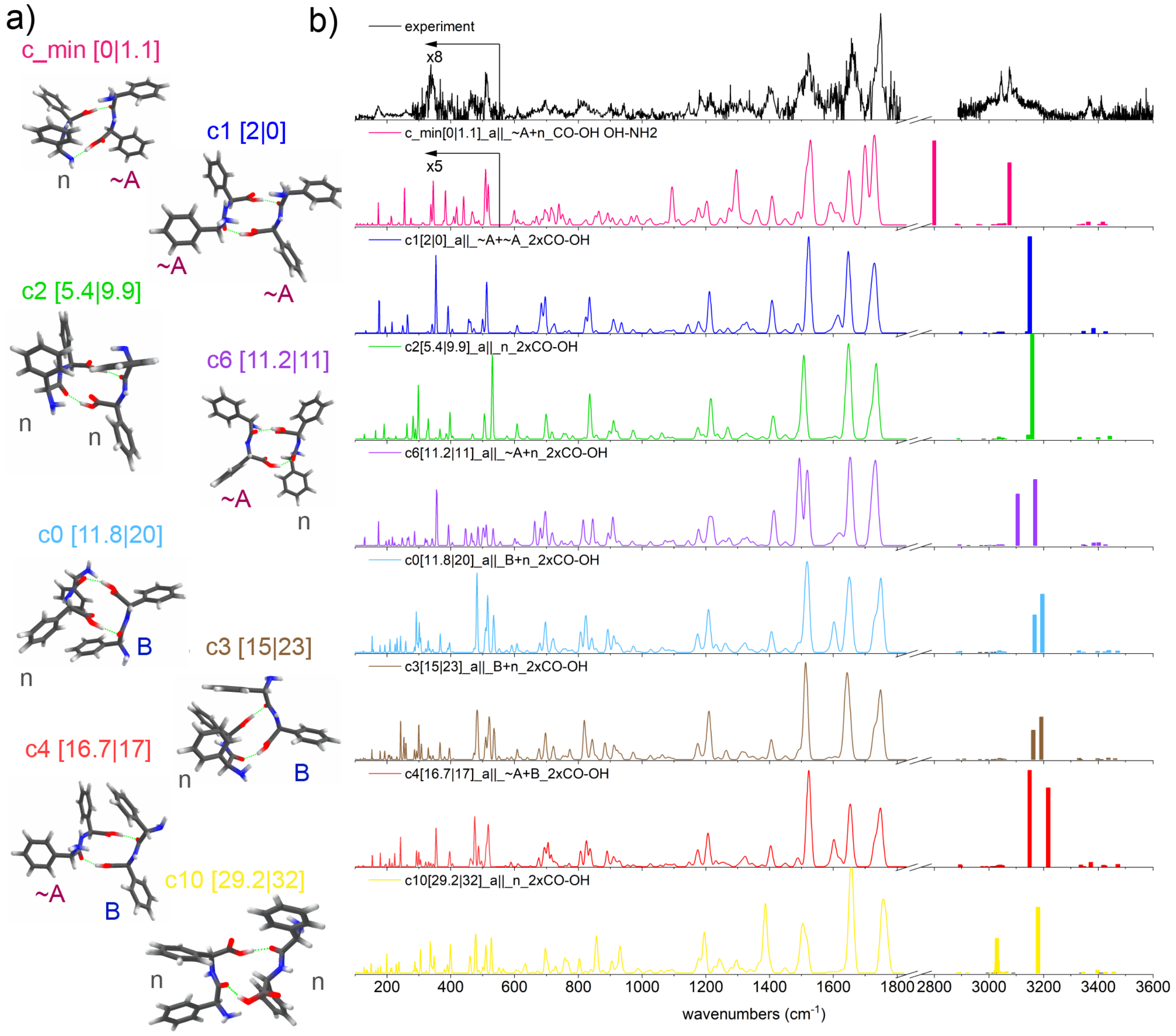

Figure S3: Theoretical IR spectra of the antiparallel family of the PhgPhg dimer and their structures: (**a**) Structures of the antiparallel family of the PhgPhg dimer with indications of conformation A, B or none of them (n) of the monomer and their zero-point energies and Gibbs free energies at 300 K in kJ/mol shown between square brackets. Intermolecular hydrogen bonds are shown in green dashed lines. (**b**) Experimental (black, top) and calculated (colored) IR spectra of the lowest energy structure (pink) and structures from the antiparallel 2xCO-OH family of the PhgPhg dimer, arranged from lowest energy to highest (top to bottom). Calculated spectra are scaled by 0.976 (1800-100 cm^{-1}) and 0.956 (2900-3600 cm^{-1}), calculations have been done at the B3LYP-D3/6-311+G(d,p) level. The region below 570 cm^{-1} was multiplied for clarity by 8 for the experimental data and by 5 for all calculated spectra.

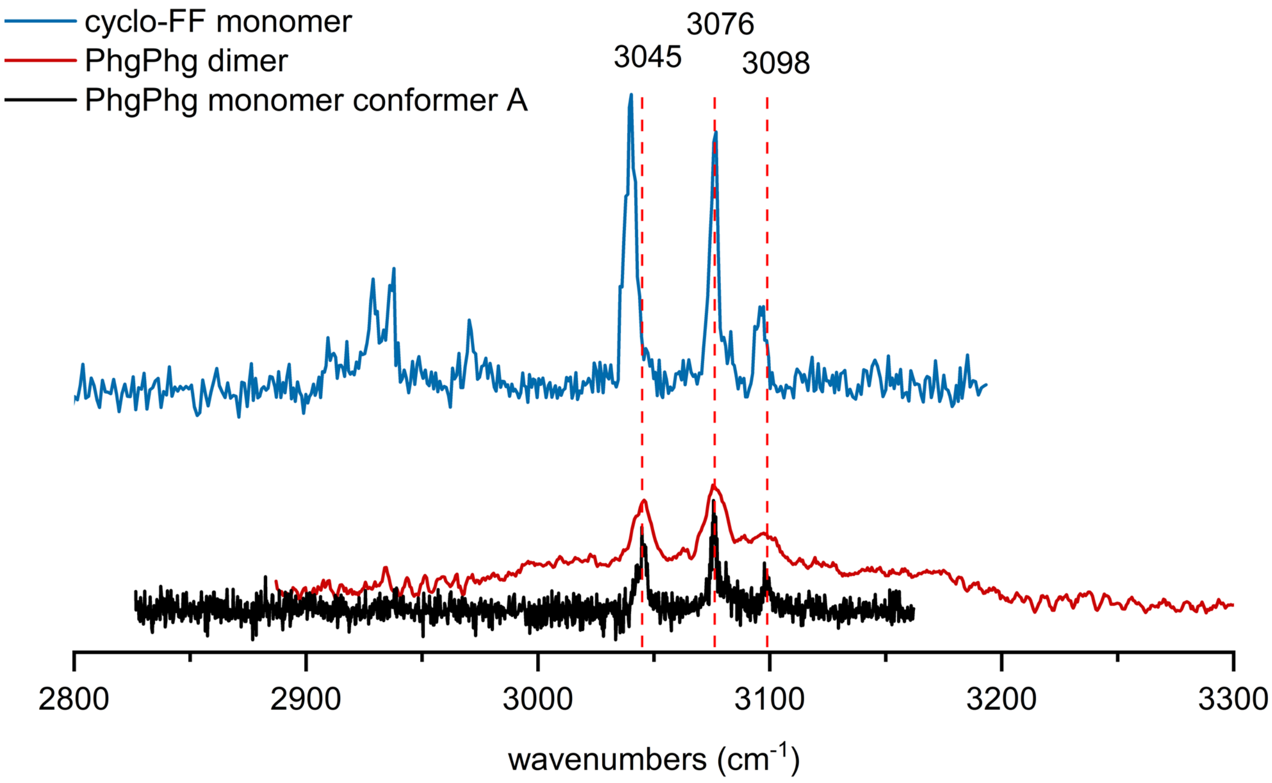

Figure S4: Comparison of IR spectra in the 3 μm region. IR spectra of the cyclo-FF monomer (dominant conformer, blue), the PhgPhg monomer (conformer A, black) and dimer (red) in the 3 μm region. IR spectrum of cyclo-FF monomer was recorded at the UV wavelength of 37588 cm^{-1}. The red dashed lines indicate the positions of the peaks that are present in the spectra of all molecules.

5. Theoretical IR frequencies characteristic for the PhgPhg dimer

Table S2: Experimentally observed (bold) and calculated frequencies in the amide I and II region for the PhgPhg dimer[1]

**Carboxylic C=O stretch**		**Peptide C=O stretch**		**NH bend**		**NH_2 scissoring**	
Free<br>OH is free	1776-1760	Free	1700-1685	Free	1530-1495	Free	1630-1624
Free<br>OH is HB	1766-1720	HB<br>C=O···OH<br>intra	1665-1644			NH_2 – π interaction	1645-1620
HB<br>C=O···OH<br>inter	1722 (w)[2]<br>1693 (s)	HB<br>C=O···OH<br>inter	1653-1630	HB<br>NH···C=O<br>inter	1560-1525	NH_2···NH<br>HB intra	1627-1614
HB<br>C=O···NH<br>inter	1747 (w)<br>1713 (s)	HB<br>C=O···NH<br>inter	1660-1653			NH_2···C=O<br>HB intra	1600-1579
						NH_2···OH<br>HB inter	1632-1609
**Experiment**	**1748**	**Experiment**	**1660**	**Experiment**	**Broad, maximum at 1522**	**Experiment**	**Broad, max at 1607**

[1]Frequencies are given in cm^{-1}. Calculated frequencies are scaled by 0.976.
[2]The (w) means weak hydrogen bond, while (s) refers to the group involved in a strong hydrogen bond. Free means no intermolecular hydrogen bonds, though the group can have intramolecular interactions within the monomer unit.

6. REMPI spectra of the FF monomer and dimer and IR-UV hole-burning spectrum of the FF monomer

The REMPI spectrum of the FF monomer has already been reported by Abo-Riziq et al. [1] and Pérez-Mellor et al. [2]. The electronic spectrum shown in this paper is similar to those reported earlier, however the positions of the peaks are slightly shifted probably due to different laser calibrations. The presence of two conformations of the monomer was reported in both papers. In this work, the existence of the second conformer (B, marked with purple asterisks) was also confirmed using IR-UV hole-burning spectroscopy (see Figure S5b). The corresponding excitation UV wavenumbers selected for the IR-UV ion dip spectroscopy experiment are marked with the pink (conformer A) and red (conformer B) asterisks in Figure S5a. The IR spectrum of conformer B was recorded in between two peaks corresponding to the UV transitions of two chromophores of conformer B. The REMPI spectrum of the dimer of FF is shown in Figure S5c with the blue asterisks indicating the position of the UV wavenumbers chosen to obtain the IR spectrum. The resulting IR spectra of the dimer are all the same, however considering the broadness of both the UV and IR spectra the presence of multiple conformations for the FF dimer cannot be excluded.

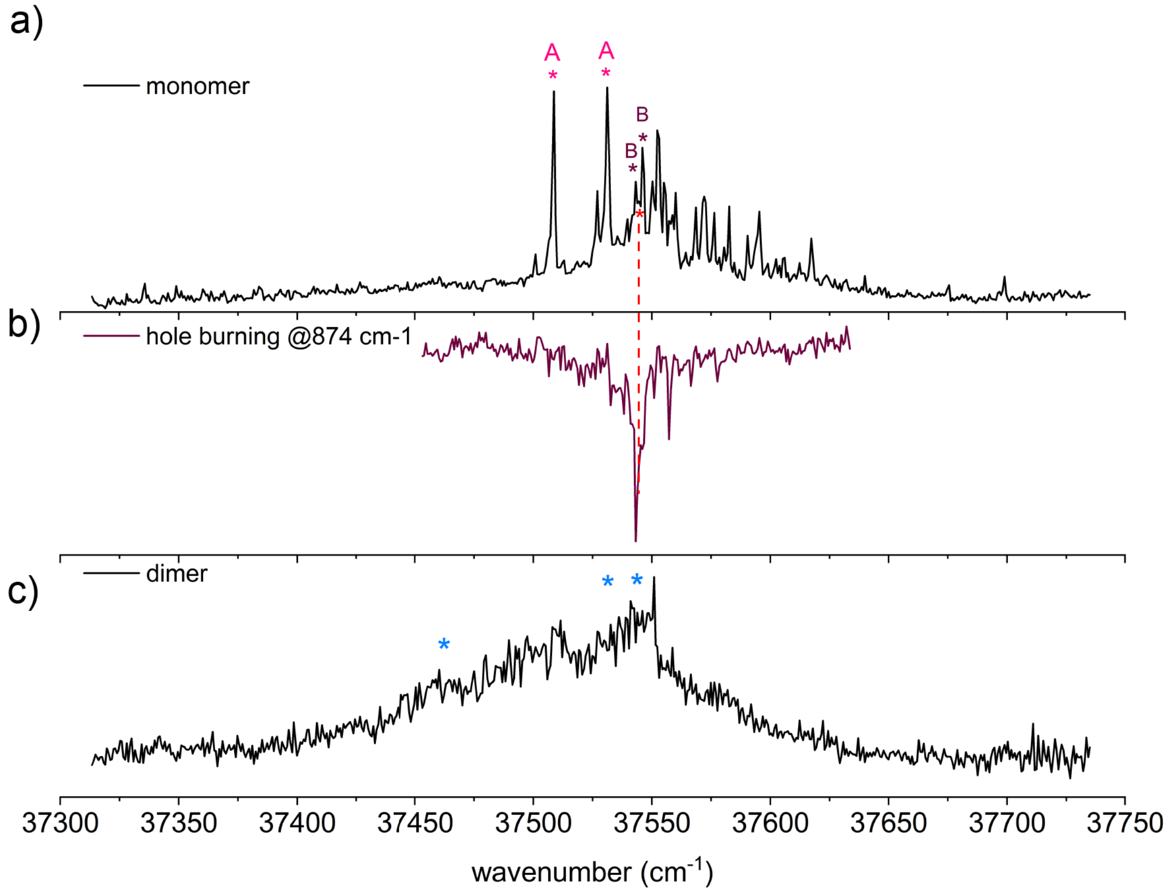

Figure S5: REMPI spectra of the monomer **(a)** and dimer of FF **(c)**, and hole-burning spectrum of the monomer **(b)**. The UV excitation wavenumber to record the IR spectrum of conformation A is marked with the pink asterisks (37508 and 37532 cm^{-1}) and for conformer B with the red asterisk (at 37544 cm^{-1}). The IR-UV hole-burning spectrum was recorded with the IR laser set at 874 cm^{-1} corresponding to the unique vibrational transition of the conformer B. The blue asterisks indicate the UV wavelengths that were chosen for recording the IR spectrum of the dimer (37462, 37532, and 37544 cm^{-1}).

7. The structural families of the FF monomer and their IR spectra

DFT calculations resulted in multiple structures of the FF monomer with different hydrogen bond patterns. Among them, the most stable variants with characteristic hydrogen bonds were summarized into four families (see Figure S6a). The types of the families are similar to the ones that were presented for the PhgPhg monomer. The I family has two hydrogen bonds with the NH group interacting with the carboxylic OH and the N-terminal NH_2 group, while the OH group remains free. The II family is characterized by the OH group hydrogen bonded to the peptide C=O. The III family has two hydrogen bonds between the peptide C=O···NH and the carboxylic C=O···NH_2 with two aromatic rings pointing to different sides of the peptide backbone. The IV family has its OH group hydrogen bonded with NH, and the peptide C=O interacting with NH_2. The families are listed by their lowest energy structures from lowest (I) in energy to the highest (IV).

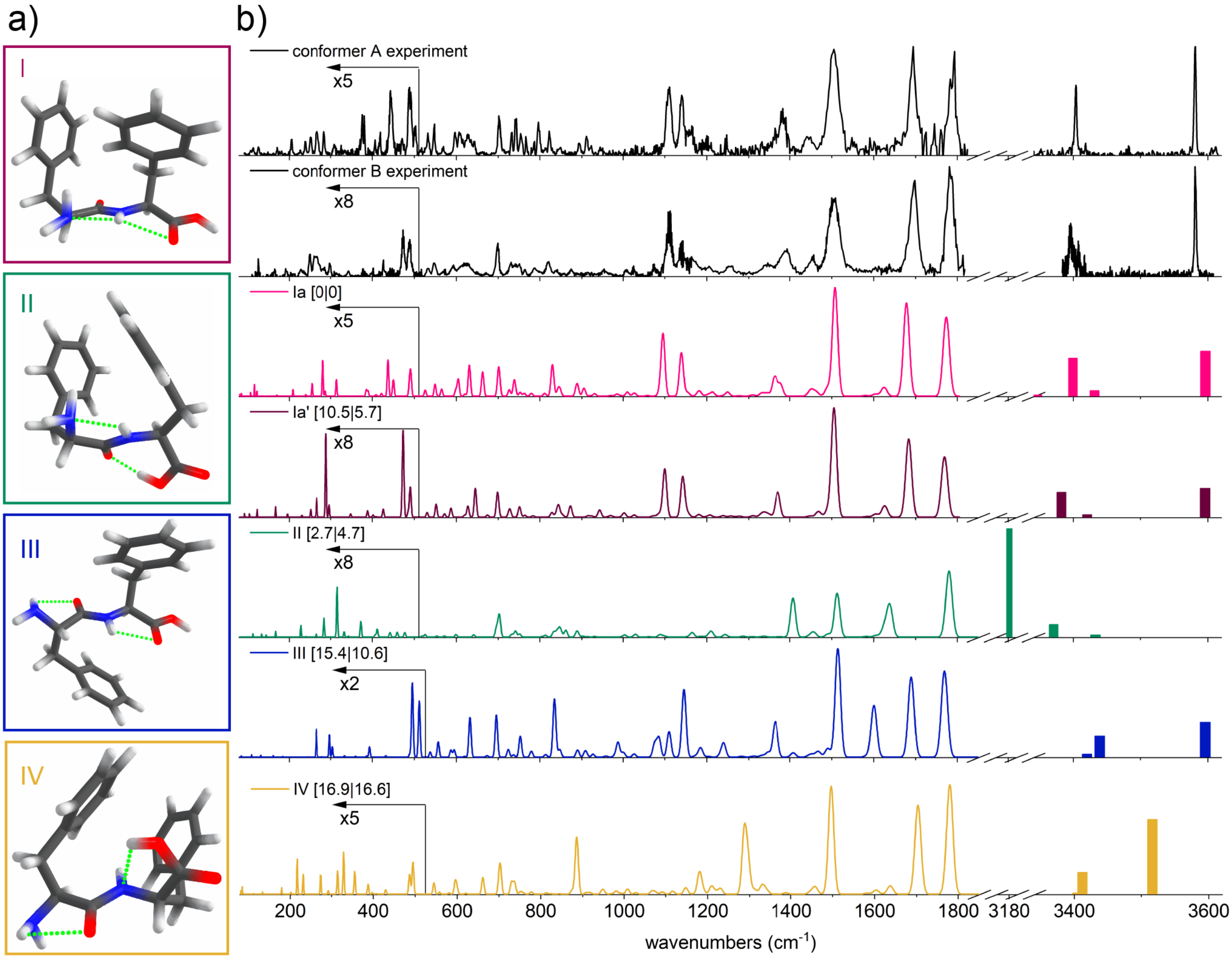

Figure S6: The structural families of the FF monomer and their IR spectra: (**a**) The structural families of the FF monomer. (**b**) The experimental spectrum of the conformers A and B of the monomer (black, top) and the calculated IR spectra of the assigned structures (pink and purple); and the theoretical spectra of the rest of the families, arranged from lowest energy to highest (top to bottom) with their zero-point energies and Gibbs free energies at 300 K in kJ/mol shown between square brackets. Calculated spectra are scaled by 0.976 (1800-100 cm^{-1}) and 0.956 (2900-3600 cm^{-1}), calculations have been done at the B3LYP-D3/6-311+G(d,p) level. The region below 520 cm^{-1} was multiplied for clarity with multiplication factors indicated.

The experimental IR spectra of both conformations of the FF monomer are shown in Figure S6b (top, black). The amide A region has been reported previously by Abo-Riziq et al. [1] and Pérez-Mellor et al. [2]. Here, we will describe the IR pattern in the 3 μm region, as well as in the mid and far-IR region between 1800 and 100 cm^{-1} which extends the analysis. Additionally, the differences and similarities of the structural assignments will be discussed. Conformation A has three peaks at 3404, 3418 and 3581 cm^{-1}. All frequencies are slightly blueshifted in about 10 cm^{-1} compared to the

ones reported previously, probably due to the laser calibration, however the pattern remains similar. The first peak at 3404 cm^{-1} can be assigned to the weakly hydrogen bonded NH group, while the second weak transition at 3418 cm^{-1} originates from an NH_2 asymmetric stretch, and the third peak at 3581 cm^{-1} belongs to a free OH stretch. The experimental spectrum of conformer B has four transitions in the amide A region and the peaks are slightly broader. The first peak at 3396 cm^{-1} corresponds to the weakly hydrogen bonded NH group, the second small peak at 3408 cm^{-1} originates from the NH_2 asymmetric stretch, while the third peak at 3416 cm^{-1} hasn't been reported earlier. This vibration possibly belongs to a combination band or an overtone. The last peak at 3582 cm^{-1} is a typical peak of the free OH stretching vibration. The position of this OH band around 3580 cm^{-1} for both conformers allows us to exclude the OH hydrogen bonded structures from the II and IV families. The III family that is characterized by the free OH group predicts the OH vibration well, however in the NH region around 3400 cm^{-1} the position of the peaks does not match with the experimental values. The NH_2 asymmetric stretch is more red shifted and has a lower energy than the NH stretching mode. Therefore, the III family can be also excluded and therefore both conformers should belong to the I structural family. The mid- and far-IR regions can confirm this and provide a more detailed structural assignment.

The experimental spectra of the conformations A and B are very similar in the 1800-1000 cm^{-1} region. The carboxylic C=O stretch vibration is located at around 1780 cm^{-1} for both conformers and suggests that the C=O can be involved in a weak C5 interaction. The peptide C=O peak is present at 1695 cm^{-1}, which is typical for a free C=O group. The peak at 1504 cm^{-1} is assigned for both conformers to the NH group that is involved in a weak C5 or NH-π interaction. Therefore, that region confirms that both conformers originate from the I structural family. The far-IR region below 1000 cm^{-1} gives more insight into the structural assignment, since it is sensitive to small alterations in the orientation of the functional groups and comprises global motions of the whole molecule. The two conformers are also more different in that region, especially below 500 cm^{-1}. The IR spectrum of conformer A has a broad peak around 480 cm^{-1}, followed by another quite broad peak at 444 cm^{-1}, a doublet at 412 cm^{-1}, another broad feature at 376 cm^{-1}, a quartet of peaks between 300-226 cm^{-1}, and a number of smaller peaks below 200 cm^{-1}. Conformer B has a completely different IR pattern in this region, such as a doublet at 478 cm^{-1}, followed by a number of low intensity peaks, and relatively strong transitions around 260 cm^{-1}. Based on the comparison of the pattern of IR transitions in the far-IR region, we can assign conformations A and B to their structures (pink and purple spectra respectively in Figure S6b). The conformer A is assigned to the most stable structure Ia, while the assigned structure of conformer B (Ia′) is more extended due to the aromatic rings rotated away from each other and is therefore 10.5 kJ/mol higher in energy.

The structural assignments that were reported previously by Abo-Riziq et al. [1] are exactly similar to our results, however our structures are assigned vice versa. Since they did not observed low intensity peak at 3418 cm^{-1} both in experiment for conformer A and in calculations for the Ia′ structure, they assigned the more extended structure to conformer A, and the more compact Ia to conformer B, while our results show it the other way around. This can originate from experimental limitations in sensitivity and/or calculations with lower basis sets and functional without empirical dispersion (B3LYP/6-311G(d,p)). In our work, the B3LYP-D3/6-311+G(d,p) functional and basis set were used. Additionally, Abo-Riziq et al. reported the structure from the II family with the hydrogen bonded OH group as the most stable one, however, that is different from our findings where the lowest energy structure originates from the I family. That difference could be explained, as was already mentioned in the work of Pérez-Mellor et al. [2], due to the fact that they did not use empirical dispersion in the calculations and consequently aromatic interactions between rings were not taken into account.

The structural assignment made by Pérez-Mellor et al. [2] coincides well with our results. They used the same dispersion-corrected functional B3LYP-D3 with an even higher 6-311++g(d,p) basis set, which includes diffuse functions also for hydrogen. Conformer A is assigned to the most stable structure which is exactly the same as our assignment. Conformer B is assigned to the more extended structure which is very similar to our assignment, except for the rotated COOH group by 180°. Figure S7 shows the comparison between the experimental IR spectrum of conformer B and the two structures Ia′ and Ib which differ only in the orientation of the COOH group and are calculated at the level of theory that we used in the current work. Two theoretical spectra are different in the amide A region, where the predicted frequency of the NH stretch is shifted from the experimental value to the red in the Ia′ structure and to the blue in the Ib structure. However, the spacing between two peaks of about 10 wavenumbers corresponding to NH and NH_2 stretch vibrations is predicted better in Ib structure. Based on the spectra only in the 3 μm region, Ib looks like a possible assignment for conformer B, however mid- and far-IR regions help us to make a more precise assignment. In the amide I and II region both spectra are equal, however Ia′ predicts the doublet at about 1400 cm^{-1} better, as well as another doublet at 1100 cm^{-1} in terms of ratio of the two peaks. Only a small peak at 1073 cm^{-1} is predicted better by Ib than by Ia′. The doublet around 1020 cm^{-1} is also described better by structure Ia′, along with another small peak at 950 cm^{-1}.

Below 950 cm-1 the two calculated structures become almost the same, except for the doublet at 485 cm-1, which again Ia′ describes better than Ib in terms of relative intensities. Therefore, based on the comparison of experimental and theoretical spectra in the mid- and far-IR region, we confidently assign conformer B to the Ia′ structure, which also is lower in energy than Ib.

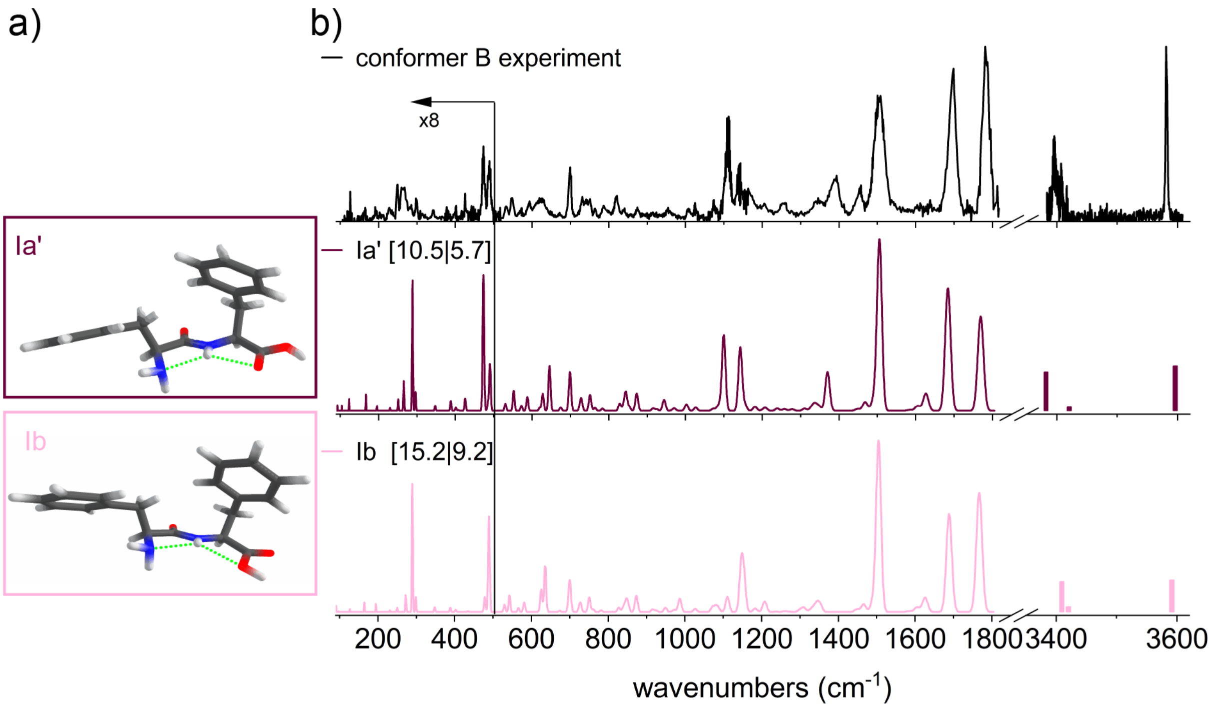

Figure S7: Comparison with previous assignment for conformer B of the FF monomer: (**a**) The calculated structures Ia′ and Ib. (**b**) The experimental spectrum of conformer B of the monomer (black, top) and the calculated IR spectra of the two structures (purple and pink) with their zero-point energies and Gibbs free energies at 300 K in kJ/mol shown between square brackets. Calculated spectra are scaled by 0.976 (1800-100 cm-1) and 0.956 (3300-3600 cm-1), calculations have been done at the B3LYP-D3/6-311+G(d,p) level. The region below 520 cm-1 was multiplied for clarity by 8 for all spectra.

8. Theoretical IR frequencies characteristic for the FF dimer

Table S3: Experimentally observed (bold) and calculated frequencies in the amide I and II region for the FF dimer[1]

Carboxylic C=O stretch		Peptide C=O stretch		NH bend		NH_2 scissoring	
Free<br>OH is free	1770-1740	Free	1693-1670	Free	1527-1507	Free	1635-1626
Free<br>OH is HB	1755-1710	HB<br>CO···NH_2	1669-1662	HB<br>NH···NH_2	1573-1543	NH_2···CO<br>intra/inter	1605/<br>1648-1606
HB<br>C=O···OH<br>inter	1743-1720	HB<br>C=O···OH<br>inter	1653-1630	HB<br>NH···C=O<br>inter	1550-1530	NH_2···NH<br>intra/inter	1625/<br>1658-1607
HB<br>C=O···NH<br>inter	1752-1695	HB<br>C=O···NH<br>inter	1675-1650			NH_2···OH<br>inter	1611-1603
**Experiment**	**1747**	**Experiment**	**1691<br>1654**	**Experiment**	**Broad, max at 1522**	**Experiment**	**Broad, max at 1614**

[1]Frequencies are given in cm^{-1}. Calculated frequencies are scaled by 0.976.

9. Theoretical IR spectra of the structural families of the FF dimer

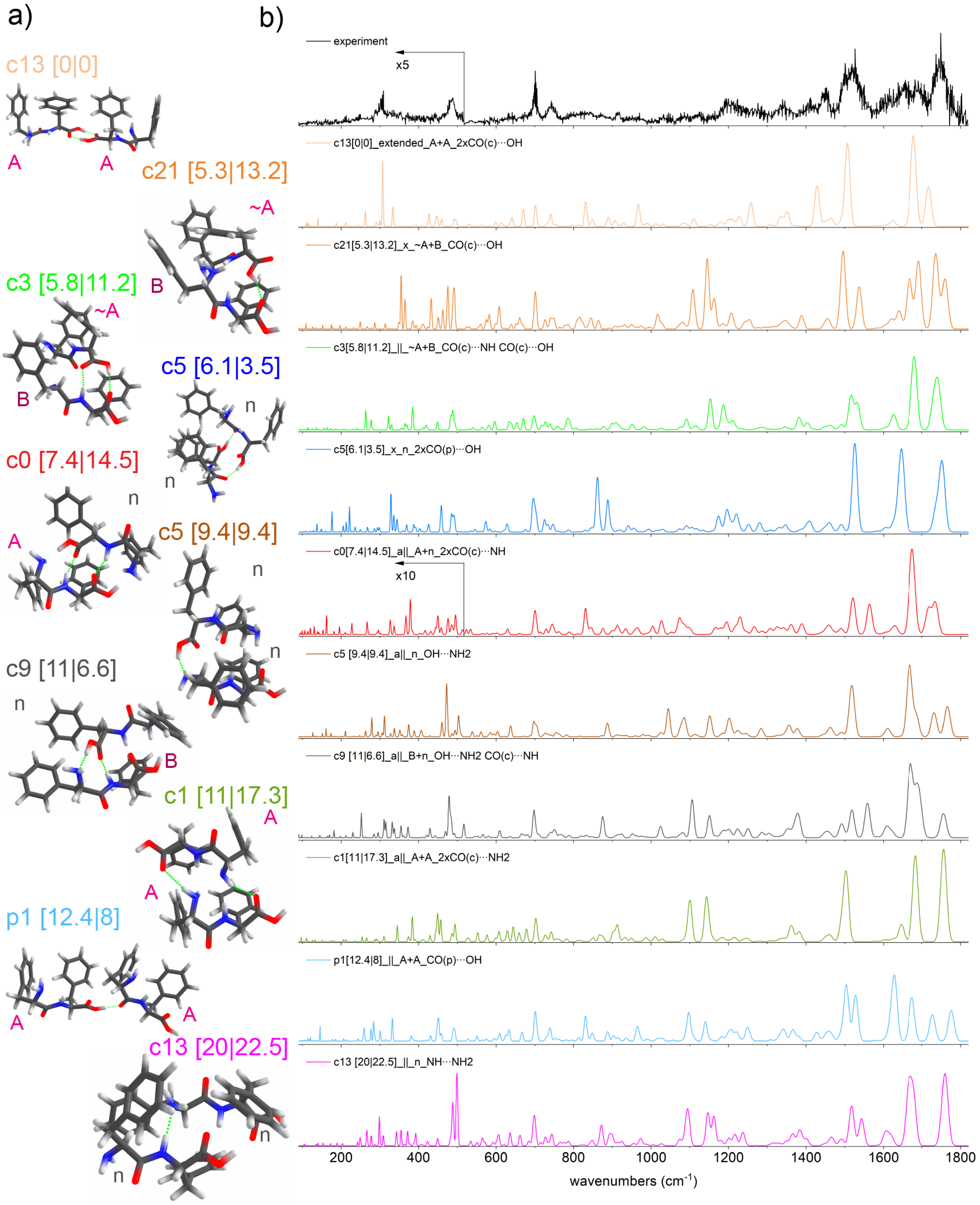

Figure S8.1: Theoretical IR spectra of the structural families of the FF dimer: (**a**) The structures of the FF dimer with an indication of the monomeric backbones originating from conformation A, B or none of them (n) and their zero-point energies and Gibbs free energies at 300 K in kJ/mol shown between square brackets. Intermolecular hydrogen bonds are shown in green dashed lines. (**b**) Experimental (black, top) and calculated (colored) IR spectra of the structures, arranged from lowest energy to highest (top to bottom). The types of intermolecular hydrogen bonds are named in the legend, as well as parallel (||), antiparallel (a||) or random (x) orientation of monomers with respect to each other. Calculated spectra are scaled by 0.976 (1800-100 cm^{-1}), calculations have been done at the B3LYP-D3/6-311+G(d,p) level. The region below 520 cm^{-1} was multiplied for clarity by 5 for all spectra and by 10 for c0 spectra (red).

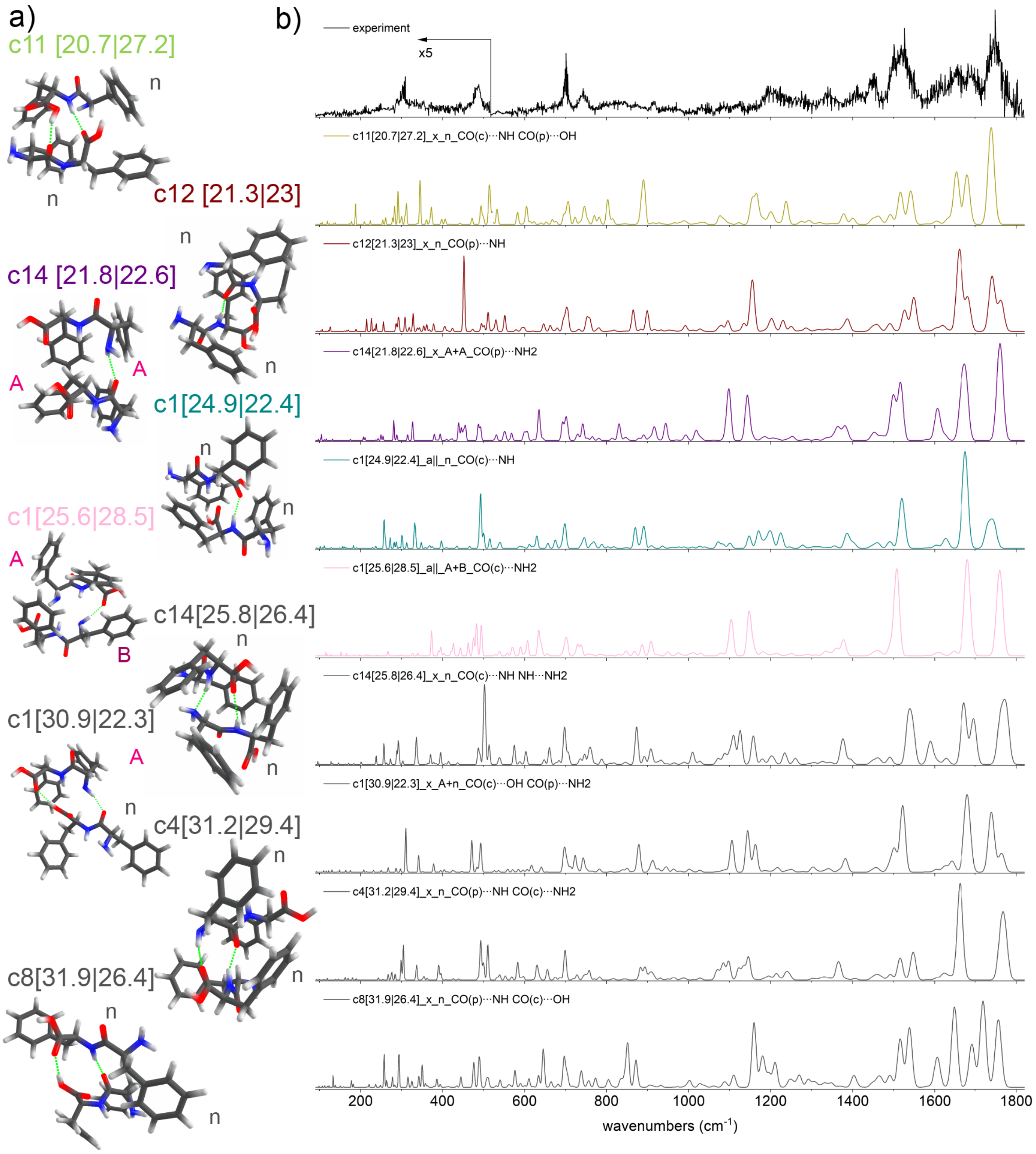

Figure S8.2: Theoretical IR spectra of the structural families of the FF dimer: (**a**) The structures of the FF dimer with an indication of the monomeric backbones originating from conformation A, B or none of them (n) and their zero-point energies and Gibbs free energies at 300 K in kJ/mol shown between square brackets. Intermolecular hydrogen bonds are shown in green dashed lines. (**b**) Experimental (black, top) and calculated (colored) IR spectra of the structures, arranged from lowest energy to highest (top to bottom). The types of intermolecular hydrogen bonds are named in the legend, as well as parallel (||), antiparallel (a||) or random (x) orientation of monomers with respect to each other. Calculated spectra are scaled by 0.976 (1800-100 cm^{-1}), calculations have been done at the B3LYP-D3/6-311+G(d,p) level. The region below 520 cm^{-1} was multiplied for clarity by 5.

10. REMPI spectrum of the FFF dimer

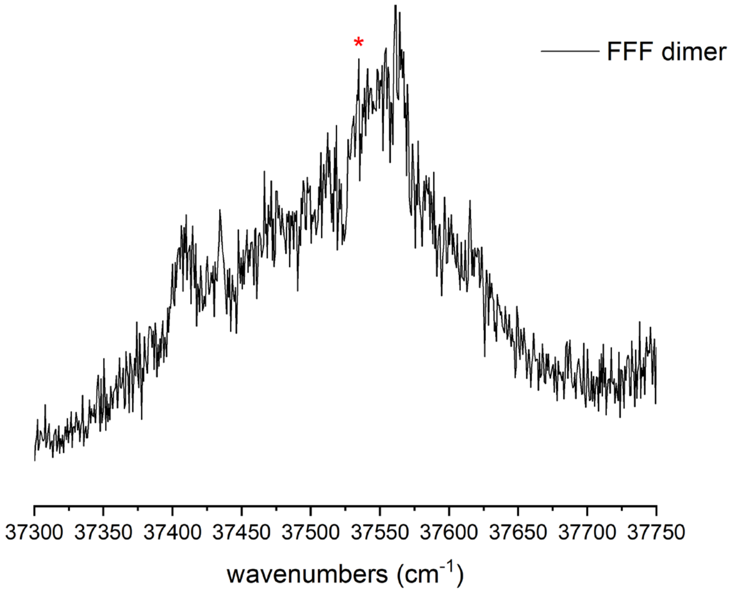

Figure S9: REMPI spectrum of the FFF dimer. The red asterisk indicates the position of UV excitation wavelenght (37535 cm^{-1}) that was chosen to record the IR spectrum.

11. Possible structure of the FFF dimer

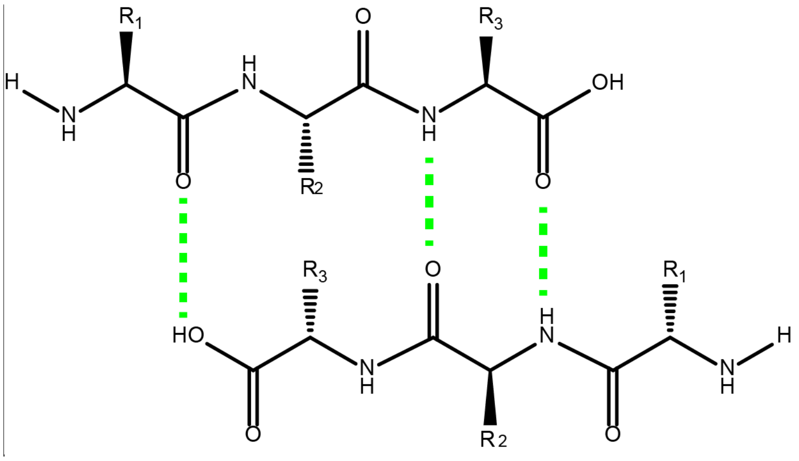

Figure S10: Possible structure of the FFF dimer. Intermolecular hydrogen bonds are shown in green dashed lines.

12. XYZ files of assigned structures

PhgPhg monomer, conformer A

#P B3LYP/6-311+G** EmpiricalDispersion=GD3 Guess=Always freq=noraman Opt=tight int=ultrafine

```
N       -1.855159000     -2.900538000      0.167648000
H       -2.785973000     -3.087721000      0.526005000
H       -1.518079000     -3.749152000     -0.274490000
C       -1.881617000     -1.791782000     -0.789998000
C       -3.472724000      1.658359000     -0.523135000
C       -2.961087000      0.472624000     -1.042178000
C       -2.520137000     -0.544390000     -0.189014000
C       -2.598965000     -0.352786000      1.193548000
C       -3.117469000      0.833774000      1.712806000
C       -3.553363000      1.843206000      0.857391000
H       -3.804956000      2.440243000     -1.196710000
H       -2.876642000      0.339446000     -2.114796000
H       -2.241334000     -1.124647000      1.864213000
H       -3.173692000      0.968953000      2.787338000
H       -3.953085000      2.766451000      1.261592000
H       -2.394103000     -2.028941000     -1.729408000
C       -0.450208000     -1.385555000     -1.201981000
O       -0.212841000     -0.912216000     -2.300886000
N        0.475645000     -1.526852000     -0.220293000
H        0.189630000     -1.931402000      0.663368000
C        1.788680000     -0.932549000     -0.335122000
C        0.805214000      2.639348000      0.633047000
C        0.793327000      1.250323000      0.520522000
C        1.787504000      0.596730000     -0.207853000
C        2.796711000      1.344358000     -0.817546000
C        2.811583000      2.731776000     -0.700180000
C        1.814737000      3.383515000      0.026160000
H        0.017959000      3.135608000      1.189118000
H       -0.007034000      0.683671000      0.979210000
H        3.568573000      0.840175000     -1.388404000
H        3.594871000      3.304309000     -1.184186000
H        1.821307000      4.464443000      0.110185000
H        2.217459000     -1.193281000     -1.305424000
C        2.670355000     -1.521270000      0.759895000
O        3.978206000     -1.348546000      0.483741000
H        4.486147000     -1.678319000      1.240445000
O        2.264673000     -2.053260000      1.762431000
```

PhgPhg monomer, conformer B

#p B3LYP/6-311+G** freq=noraman Opt=tight EmpiricalDispersion=GD3

```
N       -2.435030000     -2.782633000      0.826968000
H       -3.128436000     -3.313932000      0.312891000
H       -1.742018000     -3.423195000      1.202375000
C       -1.773674000     -1.815504000     -0.046758000
C       -4.289293000      1.032260000      0.307226000
C       -3.510057000     -0.083789000      0.599613000
C       -2.675521000     -0.639608000     -0.376556000
C       -2.644859000     -0.061953000     -1.649603000
C       -3.423666000      1.057607000     -1.942902000
C       -4.246946000      1.608283000     -0.963346000
H       -4.930633000      1.455610000      1.072312000
H       -3.542733000     -0.543500000      1.579865000
H       -2.006414000     -0.490549000     -2.416292000
H       -3.390025000      1.493933000     -2.934913000
H       -4.853931000      2.478011000     -1.188010000
H       -1.440755000     -2.258715000     -1.001321000
C       -0.441359000     -1.420529000      0.634381000
O        0.235080000     -2.262663000      1.208529000
N       -0.053665000     -0.128314000      0.506878000
H       -0.671257000      0.559214000      0.096899000
C        1.229941000      0.319433000      1.015561000
C        3.403935000      0.349001000     -2.122362000
C        2.367794000      0.610440000     -1.230248000
C        2.378845000      0.043126000      0.047261000
C        3.431404000     -0.790382000      0.421141000
```

C	4.469247000	-1.052863000	-0.472301000
C	4.458314000	-0.483147000	-1.743825000
H	3.390094000	0.793929000	-3.110987000
H	1.546908000	1.255591000	-1.525925000
H	3.429389000	-1.247554000	1.403983000
H	5.280826000	-1.708002000	-0.176633000
H	5.264547000	-0.688714000	-2.438882000
H	1.433115000	-0.196853000	1.955506000
C	1.143098000	1.814340000	1.288188000
O	2.121898000	2.219682000	2.118284000
H	2.054735000	3.182432000	2.208691000
O	0.326051000	2.556561000	0.802025000

PhgPhg dimer

#p B3LYP/6-311+G** freq=noraman Opt=tight EmpiricalDispersion=GD3

N	2.346964000	1.203792000	2.149564000
H	2.657343000	1.671158000	2.994393000
H	1.335832000	1.284767000	2.087421000
C	2.949727000	1.828224000	0.967031000
C	6.473466000	3.232745000	0.593204000
C	5.091728000	3.080022000	0.500193000
C	4.453648000	1.999748000	1.115879000
C	5.222120000	1.069328000	1.822981000
C	6.603326000	1.225390000	1.921107000
C	7.233362000	2.306415000	1.306028000
H	6.955338000	4.075946000	0.111107000
H	4.503070000	3.798205000	-0.059892000
H	4.736341000	0.223674000	2.294727000
H	7.188098000	0.498955000	2.474492000
H	8.308276000	2.426060000	1.381671000
H	2.507712000	2.798866000	0.719804000
C	2.670958000	0.961644000	-0.272700000
O	2.590998000	1.468955000	-1.396787000
N	2.550046000	-0.352928000	-0.025189000
H	2.488545000	-0.606971000	0.955453000
C	2.130710000	-1.317449000	-1.031208000
C	3.598272000	-4.610196000	0.225133000
C	2.813317000	-3.462988000	0.138909000
C	2.949528000	-2.595827000	-0.950051000
C	3.874131000	-2.895575000	-1.951971000
C	4.657110000	-4.045575000	-1.867165000
C	4.521831000	-4.904199000	-0.777852000
H	3.485383000	-5.277345000	1.072307000
H	2.084370000	-3.244554000	0.911634000
H	3.984298000	-2.225878000	-2.797920000
H	5.371768000	-4.269217000	-2.651101000
H	5.130509000	-5.798861000	-0.711473000
H	2.267140000	-0.850380000	-2.004878000
C	0.637104000	-1.614894000	-0.844780000
O	0.009911000	-1.782979000	-1.999572000
H	-0.968852000	-1.852348000	-1.846708000
O	0.113958000	-1.679701000	0.250101000
N	-2.346973000	-1.203790000	2.149563000
H	-1.335840000	-1.284765000	2.087425000
H	-2.657356000	-1.671154000	2.994392000
C	-2.949730000	-1.828224000	0.967028000
C	-6.473461000	-3.232765000	0.593203000
C	-5.091724000	-3.080035000	0.500193000
C	-4.453651000	-1.999755000	1.115874000
C	-5.222129000	-1.069334000	1.822970000
C	-6.603333000	-1.225402000	1.921094000
C	-7.233364000	-2.306434000	1.306021000
H	-6.955328000	-4.075971000	0.111111000
H	-4.503062000	-3.798218000	-0.059887000
H	-4.736355000	-0.223675000	2.294712000
H	-7.188110000	-0.498968000	2.474475000
H	-8.308277000	-2.426084000	1.381664000
H	-2.507710000	-2.798863000	0.719801000
C	-2.670963000	-0.961641000	-0.272701000
O	-2.591001000	-1.468951000	-1.396789000
N	-2.550054000	0.352932000	-0.025190000

H	-2.488553000	0.606973000	0.955453000
C	-2.130714000	1.317452000	-1.031207000
C	-4.657093000	4.045599000	-1.867162000
C	-3.874121000	2.895594000	-1.951971000
C	-2.949526000	2.595834000	-0.950047000
C	-2.813315000	3.462988000	0.138919000
C	-3.598264000	4.610200000	0.225145000
C	-4.521815000	4.904216000	-0.777844000
H	-5.371745000	4.269250000	-2.651102000
H	-3.984288000	2.225903000	-2.797925000
H	-2.084374000	3.244545000	0.911647000
H	-3.485374000	5.277344000	1.072323000
H	-5.130487000	5.798881000	-0.711463000
H	-2.267146000	0.850386000	-2.004878000
C	-0.637107000	1.614891000	-0.844779000
O	-0.009915000	1.782981000	-1.999572000
H	0.968848000	1.852348000	-1.846707000
O	-0.113959000	1.679690000	0.250101000

FF monomer, conformer A

B3LYP/6-311+G** EmpiricalDispersion=GD3 freq=noraman Opt=tight Guess =Always

N	-0.910473000	-1.362063000	2.207404000
H	-1.370145000	-1.641190000	3.065795000
H	-1.152903000	-0.393086000	2.021567000
C	-1.288163000	-2.229397000	1.090379000
H	-1.213467000	-3.263193000	1.446218000
C	-0.259015000	-2.127967000	-0.053355000
O	-0.502638000	-2.549727000	-1.179080000
C	-2.727212000	-2.037066000	0.536991000
H	-2.884693000	-2.776727000	-0.249693000
H	-3.424196000	-2.260813000	1.350511000
C	-2.990514000	-0.648789000	0.002159000
C	-3.417838000	0.379782000	0.851412000
H	-3.614714000	0.161870000	1.896510000
C	-2.781270000	-0.349439000	-1.351215000
H	-2.436637000	-1.133926000	-2.013978000
C	-3.615275000	1.674422000	0.370512000
H	-3.946984000	2.455826000	1.045603000
C	-2.986922000	0.940505000	-1.835482000
H	-2.818362000	1.152877000	-2.885422000
C	-3.397681000	1.959383000	-0.975707000
H	-3.548853000	2.964487000	-1.352476000
N	0.915793000	-1.558240000	0.292829000
H	1.024642000	-1.243089000	1.249951000
C	1.949123000	-1.289321000	-0.678174000
H	1.991701000	-2.122805000	-1.385211000
C	3.286693000	-1.210818000	0.035354000
O	3.450687000	-1.230708000	1.227693000
C	1.656719000	0.003998000	-1.500616000
H	2.457934000	0.132404000	-2.231478000
H	0.732295000	-0.191193000	-2.047484000
C	1.512374000	1.232106000	-0.634336000
C	0.263999000	1.593424000	-0.115131000
H	-0.614624000	1.020808000	-0.384983000
C	2.627781000	2.005715000	-0.293185000
H	3.601094000	1.744042000	-0.695901000
C	0.134531000	2.695282000	0.727884000
H	-0.845062000	2.961242000	1.108890000
C	2.502235000	3.106703000	0.551707000
H	3.377319000	3.695345000	0.804098000
C	1.254129000	3.453336000	1.067670000
H	1.154837000	4.311637000	1.722750000
O	4.305053000	-1.095282000	-0.849726000
H	5.126589000	-1.037806000	-0.339179000

FF monomer, conformer B

#p B3LYP/6-311+G** freq=noraman Opt=tight EmpiricalDispersion=GD3

N	-1.278615000	-1.456122000	1.377246000
H	-2.132520000	-1.065295000	1.761624000
H	-1.182194000	-2.396010000	1.746984000

C	-1.321709000	-1.463350000	-0.087119000
H	-1.942941000	-2.267049000	-0.503334000
C	0.073667000	-1.671407000	-0.693093000
O	0.212143000	-2.018306000	-1.860920000
C	-1.870585000	-0.115587000	-0.612177000
H	-1.232226000	0.686471000	-0.232126000
H	-1.781097000	-0.127387000	-1.701181000
C	-3.305321000	0.123701000	-0.205975000
C	-3.622352000	0.996385000	0.840339000
H	-2.825899000	1.537504000	1.341465000
C	-4.347904000	-0.557318000	-0.846978000
H	-4.117916000	-1.233303000	-1.664676000
C	-4.946445000	1.183572000	1.239886000
H	-5.172992000	1.866191000	2.051454000
C	-5.670104000	-0.372269000	-0.452255000
H	-6.465047000	-0.903837000	-0.963614000
C	-5.973830000	0.498515000	0.595506000
H	-7.003403000	0.644077000	0.902195000
N	1.104025000	-1.402025000	0.139381000
H	0.884522000	-1.094293000	1.080854000
C	2.483097000	-1.475396000	-0.288147000
H	2.651499000	-2.434220000	-0.790434000
C	3.366557000	-1.452048000	0.947003000
O	2.987482000	-1.291768000	2.079080000
C	2.873381000	-0.361915000	-1.309059000
H	3.937062000	-0.468805000	-1.531551000
H	2.315053000	-0.580527000	-2.219783000
C	2.556343000	1.029941000	-0.821720000
C	1.351146000	1.643064000	-1.178818000
H	0.671428000	1.123578000	-1.845177000
C	3.430915000	1.718718000	0.026028000
H	4.376170000	1.262897000	0.302884000
C	1.021282000	2.907670000	-0.695294000
H	0.083127000	3.368160000	-0.985540000
C	3.105464000	2.983282000	0.512417000
H	3.795620000	3.503259000	1.167488000
C	1.897143000	3.580663000	0.155310000
H	1.643770000	4.565248000	0.531905000
O	4.667646000	-1.633344000	0.623350000
H	5.177479000	-1.609599000	1.446963000

13. References

1. Abo-Riziq, A.G.; Bushnell, J.E.; Crews, B.; Callahan, M.P.; Grace, L.; De Vries, M.S. Discrimination between diastereoisomeric dipeptides by IR-UV double resonance spectroscopy and ab initio calculations. *Int. J. Quantum Chem.* **2005**, *105*, 437–445, doi:10.1002/qua.20719.
2. Pérez-Mellor, A.; Alata, I.; Lepere, V.; Zehnacker, A. Chirality effects in the structures of jet-cooled bichromophoric dipeptides. *J. Mol. Spectrosc.* **2018**, *349*, 71–84, doi:10.1016/j.jms.2018.02.005.
